# Effect of low-intensity transcranial ultrasound stimulation on theta and gamma oscillations in the mouse hippocampal CA1

**DOI:** 10.3389/fpsyt.2023.1151351

**Published:** 2023-04-20

**Authors:** Zhen Li, Rong Chen, Dachuan Liu, Xizhe Wang, Wei Yuan

**Affiliations:** ^1^Department of Ophthalmology, Xuanwu Hospital, Capital Medical University, Beijing, China; ^2^Hebei Key Laboratory of Vascular Homeostasis and Hebei Collaborative Innovation Center for Cardio-Cerebrovascular Disease, The Second Hospital of Hebei Medical University, Shijiazhuang, China

**Keywords:** transcranial ultrasound stimulation, theta oscillation, gamma oscillation, CA1, behavioral state

## Abstract

Previous studies have demonstrated that low-intensity transcranial ultrasound stimulation (TUS) can eliminate hippocampal neural activity. However, until now, it has remained unclear how ultrasound modulates theta and gamma oscillations in the hippocampus under different behavioral states. In this study, we used ultrasound to stimulate the CA1 in mice in anesthesia, awake and running states, and we simultaneously recorded the local field potential of the stimulation location. We analyzed the power spectrum, phase-amplitude coupling (PAC) of theta and gamma oscillations, and their relationship with ultrasound intensity. The results showed that (i) TUS significantly enhanced the absolute power of theta and gamma oscillations under anesthesia and in the awake state. (ii) The PAC strength between theta and gamma oscillations is significantly enhanced under the anesthesia and awake states but is weakened under the running state with TUS. (iii) Under anesthesia, the relative power of theta decreases and that of gamma increases as ultrasound intensity increases, and the result under the awake state is opposite that under the anesthesia state. (iv) The PAC index between theta and gamma increases as ultrasound intensity increases under the anesthesia and awake states. The above results demonstrate that TUS can modulate theta and gamma oscillations in the CA1 and that the modulation effect depends on behavioral states. Our study provides guidance for the application of ultrasound in modulating hippocampal function.

## 1. Introduction

Low-intensity transcranial ultrasound stimulation (TUS) is a new type of brain stimulation technology (1, 2). Compared with other traditional electrical, magnetic, and optical stimulation technologies, it has the advantages of noninvasiveness, stronger directionality, better penetration depth, and more accurate target control (3–5). Therefore, TUS has attracted much attention from scholars in the field of neuroscience. Previous studies have demonstrated that TUS can modulate neuronal activity of the rodent hippocampus (6, 7). For example, ultrasound stimulation of hippocampal slices of normal mice can induce action potentials in pyramidal neurons of the CA1 area, enhance the firing rate of action potentials, and increase the intensity of local field potentials (LFPs) in the mouse hippocampus (6). It can also modulate the phase-amplitude coupling (PAC) strength of neural oscillations in the hippocampus of rats (7).

We know that theta (4–12 Hz) and gamma (30–45 Hz) oscillations from the hippocampus play an important role in information processing. Theta oscillation represents the “online” state of the hippocampus (8, 9). It plays a role in processes such as autonomy, preparation, direction, exploration, and sleep (10). Gamma oscillation, which is a collective synchronized movement of a large number of network neurons in the brain, plays a role in high-level neural network activities, such as the production of cognition and perception, attention, learning, and memory (11, 12). Therefore, it is very important to analyze theta and gamma oscillations to investigate the changes in hippocampal function caused by ultrasound stimulation. However, until now, the effects of TUS on theta and gamma oscillations in the hippocampus have remained unclear.

In the process of brain stimulation, the influence of any external stimulus depends on the nature of the stimulus and the initial state of the brain (13). Understanding the effect of the brain state on the stimulus can provide an important basis for choosing the correct stimulation scheme and parameters. The state-dependent stimulation effect has been proven in transcranial magnetic stimulation which is used in the clinic (14, 15). In ultrasound stimulation, researchers also found that the stimulation effect depends on the animal’s state, such as being under anesthesia. We know that anesthesia can alter the activity of EEG signals and brain waves from low-amplitude fast waves to high-amplitude slow waves (16, 17). Previous studies have demonstrated that enhancement or suppression of neural activity induced by TUS will be affected by the anesthesia dose. For instance, if the anesthesia dose is too high, the animal’s motion response and EEG signal of the motor cortex cannot be observed after ultrasound stimulation (18–20).

In addition, we know that neural oscillations in the hippocampus are closely related to the state. For example, the theta and gamma oscillations in the hippocampus depend on the behavioral state. Animals have different characteristics of theta oscillation during exercise and sleep states (21), and gamma oscillations usually appear in animals during rapid eye movement sleep or awake exploration states (22). However, until now, we have not determined whether TUS-evoked theta and gamma oscillations in the hippocampus depend on behavioral states, such as anesthesia, awake, and running states.

To fill these gaps, we applied ultrasound stimulation and simultaneous electrophysiological recording of mice under anesthesia, awake, and running states to explore the absolute power, relative power of theta and gamma oscillation, and PAC between theta and gamma oscillation. In addition, the relationships between changes in relative power, PAC index, and ultrasound intensity were analyzed.

## 2. Materials and methods

### 2.1. Animals and groups

Forty-two C57BL/6 mice were used in these experiments (all males, body weights 20–25 g, Beijing Vital River Laboratory Animal Technology Co., Ltd., China). All procedures were conducted according to the guidelines of the Animal Ethics and Administrative Council of Capital Medical University. The mice were housed in standard cages with a 12-h light/12-h dark cycle and given food and water *ad libitum*. Twenty-one mice were randomly divided into three groups [anesthesia group (*n* = 7), awake group (*n* = 7), and running group (*n* = 7)] to evaluate theta and gamma oscillations induced by TUS. Twenty-one mice were used to investigate the relationship between theta and gamma oscillations and ultrasound intensity in the three states [anesthesia group (*n* = 7), awake group (*n* = 7), and running group (*n* = 7)].

### 2.2. Experimental procedures

**Figure 1:**
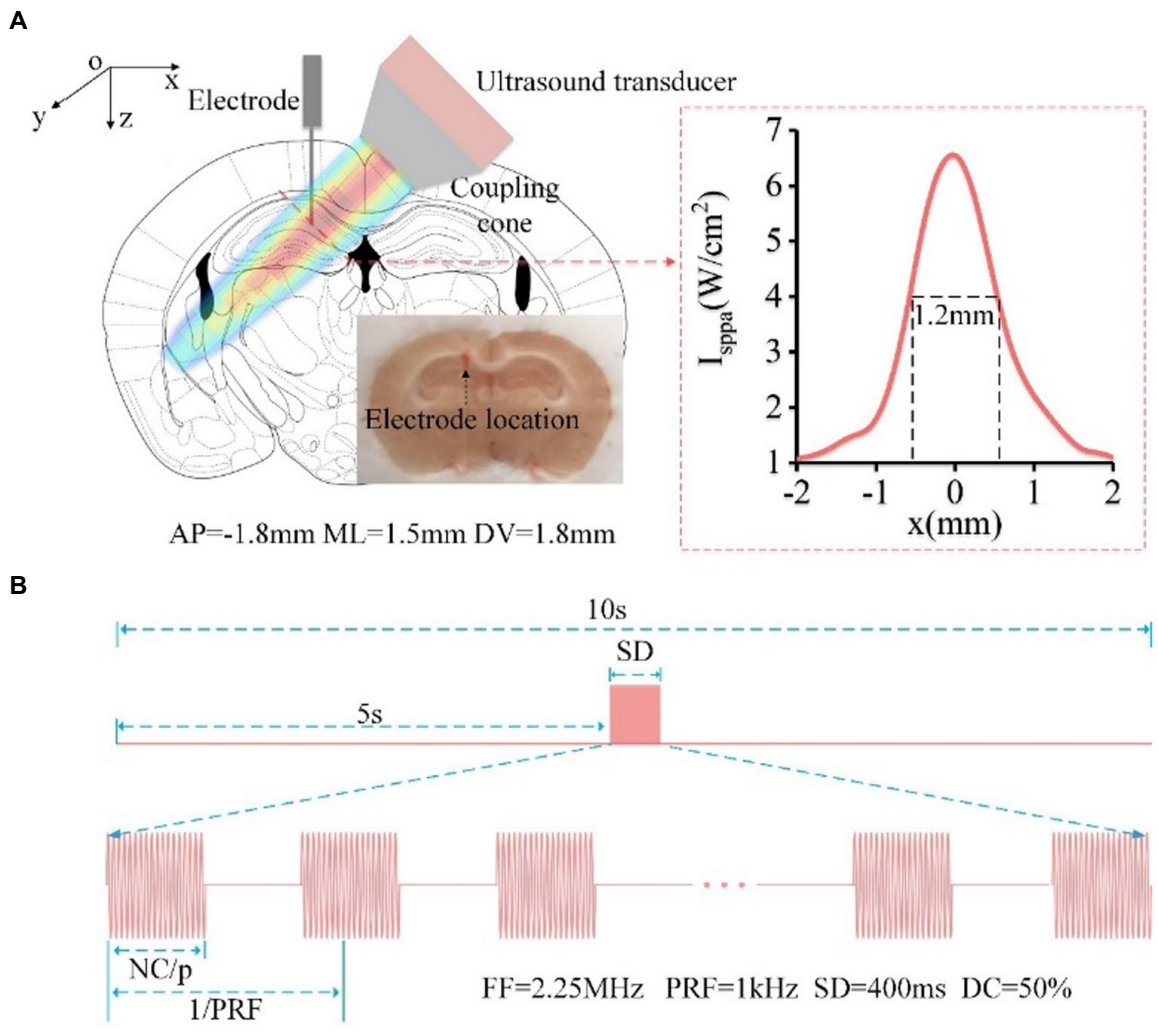
**(A)** Schematic diagram of mouse head stimulated by focused ultrasound, including the location of the ultrasound spot and electrode implantation (AP: −1.5 mm, ML: 1 mm, DV: −1.5 mm, relative to the bregma). The red dotted line in the right box is the reconstruction profile of the ultrasound field, and the FW50%M is approximately 1.2 mm. The electrode location was in CA1 area. **(B)** Timing diagram of ultrasound stimulation, FF = 2.25 MHz, PRF = 1 kHz, SD = 400 ms, DC = 50%.

We referred the methods in the literature (23, 24) to fix mouse. Self-made aluminum connecting rods was used for head-fixed. The connecting rod was connected with the mouse skull by dental cement, and fixed on the self-made experimental platform by screws. The mouse fixed on the platform stand on the treadmill. It can prevent the head of the awake mouse from moving under awake and running states. In previous study (25), this method was used for recording local field potential and action potential for awake animals. In the anesthesia group, the mice were anesthetized with 0.5% isoflurane with an oxygen delivery rate of 0.5 l/min. In the awake group, each mouse was fixed by a homemade connecting rod without anesthesia. In the running group, each mouse was fixed by a homemade connecting rod and run on a treadmill. The mice were anesthetized with 2% isoflurane for implantation of the electrode. A hole with a diameter of ~1 mm was drilled in the skull to place the recording electrode with a tip diameter of ~15 μm (CA1, AP: −1.5 mm, ML:1 mm, DV: −1.5 mm, relative to the bregma). The electrode location was in CA1 area as shown in slice in Figure 1A. Two holes were drilled in the nasal bone to fix the ground and reference electrodes.

### 2.3. TUS experimental setup and parameters

The TUS experimental setup is similar to that used in our previous paper to generate pulsed signals (26, 27). In order to ensure that the ultrasound was targeted to the CA1 area. First, we measured the distribution of ultrasound field outside the coupling cone. And then, we determined the placement of the ultrasound transducer and coupling cone based on the mouse’s brain atlas and the distribution of ultrasound field. As shown in Figure 1A, the focused ultrasound beam with an angle of ~45° was transmitted into the brain. The full width at 50% maximum (FW50%M) of the ultrasound beam in CA1 is approximately 1.2 mm. The sequence diagram of TUS and the ultrasound parameters are shown in Figure 1B. There are 16 trials for the average data in the experiment. In each trial, the fundamental frequency, stimulation duration, pulsed repetition frequency, and duty cycle were 2.25 MHz, 400 ms, 1 kHz, and 50%, respectively. The ultrasound intensities were 0.15, 0.3, 0.45, 0.6, and 0.75 MPa, and the corresponding *I*_sppa_ were 0.75, 3, 6.75, 12, and 18.75 W/cm^2^, respectively. The ultrasound intensity in the experiment for evaluating theta and gamma oscillation is 0.45 MPa.

### 2.4. Data acquisition

A multichannel data acquisition system (Apollo, Bio-Signal Technologies: McKinney, TX, U.S.A) was used to simultaneously record the trigger signal from the functional generator and LFP signals from the electrode. The sampling rate of LFP signals was 1 kHz.

### 2.5. Power spectrum analysis

The Morse Wavelets was used to calculate the time-frequency diagram of LFPs (28). The symmetry parameter and time-bandwidth product of Morse wavelet were 3 and 60, respectively. The Welch algorithm was used to analyze the power spectrum of LFPs with data segments, adding window functions, and averaging data. The stimulus start time was marked as time 0, and data from each ultrasound stimulus were divided into 10 segments: [−5–-4 s], [−4–-3 s], [−3–-2 s], [−2–-1 s], [−1–0 s], [0–1 s], [1–2 s], [2–3 s], [3–4 s], and [4–5 s]. The absolute power (AP) of the theta [4–12 Hz] and gamma [30–45 Hz] frequency bands were calculated by the Welch algorithm. The total absolute power of the frequency bands was obtained by 4–100 Hz frequency bands. The power value determined using a mean value. The relative power (RP) of each frequency band was equal to the corresponding absolute power divided by the total absolute power. When we calculated the change of AP and RP, the average values of *AP* and *RP* during 5 s before TUS was calculated as baseline (*AP*_baseline_ and *RP*_baseline_). They can be expressed by the following formular: $AP_{\mathrm{ba}seline}=\left(\overset{-1}{\underset{t=-5}{\sum }}AP_{t-t+1}\right)/5$, $RP_{\mathrm{ba}seline}=\left(\overset{-1}{\underset{t=-5}{\sum }}RP_{t-t+1}\right)/5$. The change of *AP* (Δ*AP*/*AP*) and *RP* (Δ*RP*/*RP*) was expressed as $\Delta AP/AP=\frac{AP_{t1-t2}-AP_{\mathrm{ba}seline}}{AP_{\mathrm{ba}seline}}\Delta RP/RP=\frac{RP_{t1-t2}-RP_{\mathrm{ba}seline}}{RP_{\mathrm{ba}seline}}$, where *AP*_t1–t2_ and *RP*_t1–t2_ are the values of absolute power and absolute power at time of 0–1, 1–2, 2–3, 3–4, 4–5 s.

### 2.6. PAC analysis

Phase-amplitude coupling is the index of the coupling degree between the low-frequency phase and high-frequency amplitude. In our study, a two-way, zero phase-lag, finite impulse response filter with hamming window was used for low-pass filtering of local field potential to get the low-frequency signal. The phase of low-frequency signal (denoted by *φ*_l_) was calculated by Hilbert transform. The same filter was also used for high-pass filtering of local field potential to get the high-frequency signal. The analytic amplitudes were obtained by calculating the absolute values of high-frequency signal. And then, the analytic amplitudes were filtered by the above filter with low-pass frequency band, which was the same to that for calculating *φ*_l_. The phase of the analytic amplitudes filtered by low-frequency band (denoted by *φ*_h_) was calculated by Hilbert transform. The PAC index (PACI) between the two signals was defined by the following equation (29):


(1) $$PACI=\left|\frac{1}{K}\overset{K-1}{\underset{k=1}{\sum }}\exp\left(i\left(\phi_{l}\left[k\right]-\phi_{h}\left[k\right]\right)\right)\right|$$

where PACI is the value of phase-locking between ongoing phase $\phi_{l}$ and $\phi_{k}$, and k is the time index.

### 2.7. Statistical analysis

The Kruskal–Wallis test with post-hoc pairwise comparison was used to test for statistical significance. Differences were considered to be significant when *p* < 0.05. Statistical analyses were conducted using MATLAB software.

## 3. Results

### 3.1. Power spectrum of theta and gamma oscillations induced by TUS under different behavioral states

**Figure 2:**
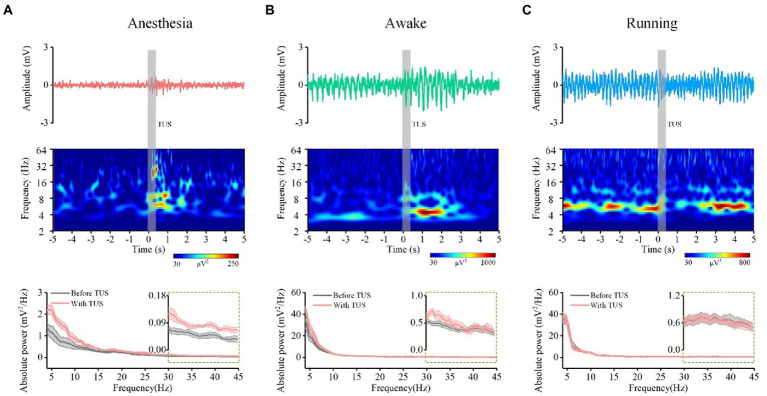
The LFP from one typical mouse (top), time-frequency diagram (middle) corresponding to the LFP, power spectrum curve (bottom, *N* = 7) of CA1 before and with ultrasound stimulation. **(A)** Anesthesia state. **(B)** Awake state. **(C)** Running state.

The absolute power spectrum of the EEG signal represents the energy change of certain frequency bands of the EEG signal, and the relative power spectrum can reflect the relationship of energy change between a certain frequency band and other frequency bands (30, 31). To evaluate the power spectrum of theta and gamma oscillations induced by TUS under different behavioral states, we first analyzed the LFP from the mouse CA1. Figures 2A–C (upper) shows the LFPs before and with TUS from a typical mouse. We can see that the amplitude of the LFP under the awake and running states is higher than that under the anesthesia state before TUS. We also notice that the amplitude of LFP with TUS increases under the anesthesia and awake states but decreases under the running state. The results of the time-frequency diagram corresponding to the LFP are shown in Figures 2A–C (middle). They indicate that the intensity of theta and gamma frequency bands are significantly enhanced within 2 s with stimulation under the anesthesia state; the intensity of theta frequency band is significantly enhanced under the awake state; the intensity of theta and gamma frequency bands does not change under the running state. We conducted statistical analyses of the power spectrum curves of all samples (*N* = 7 for each state), as shown in Figures 2A–C (bottom). The results show that the power spectrum intensity of theta and gamma frequency bands with stimulation increase under the anesthesia and awake states and is close to that before stimulation under the running state.

**Figure 3:**
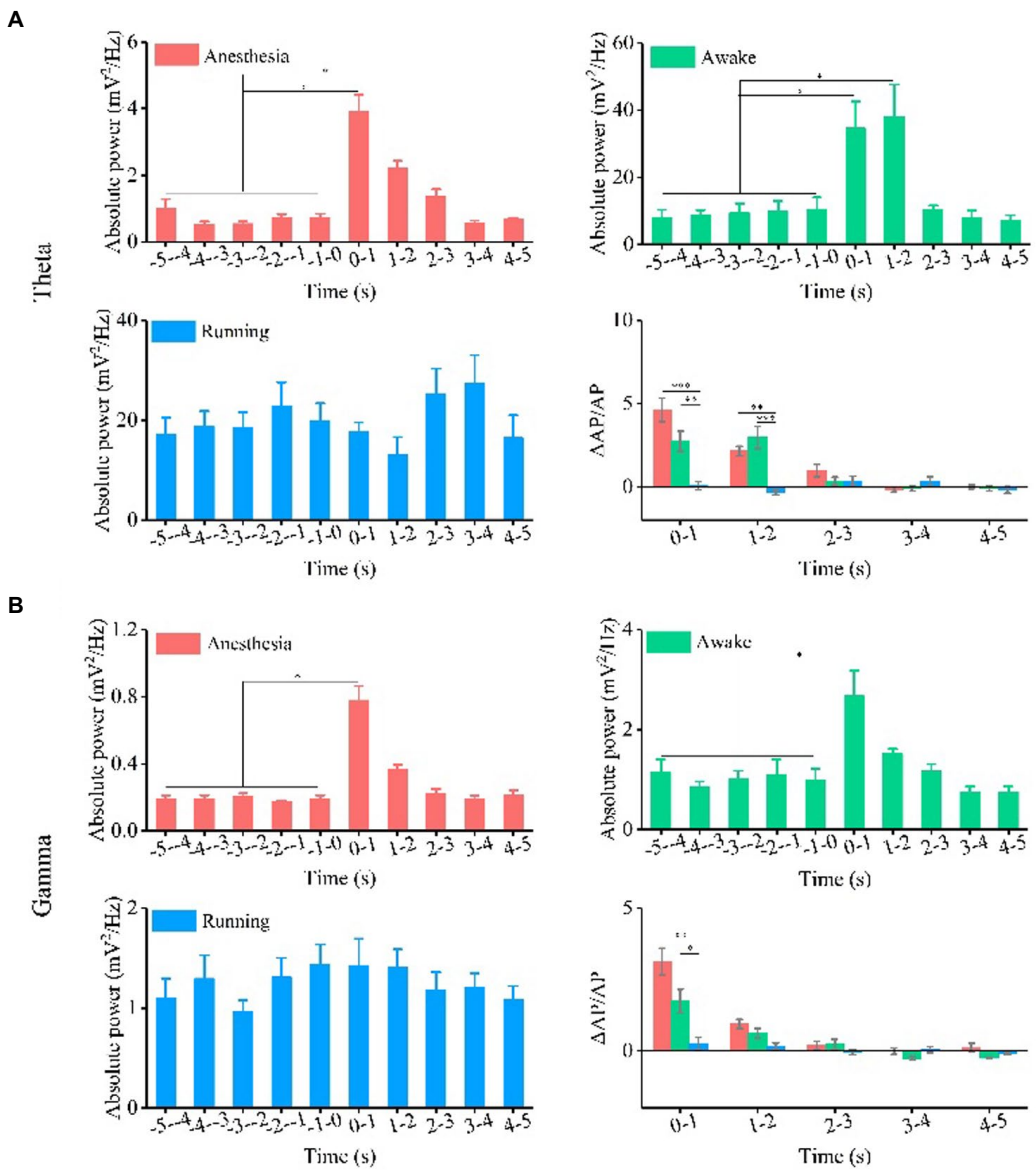
Absolute power and relative change of absolute power of theta and gamma oscillation under the anesthesia, awake state, and running state. **(A)** Theta oscillation. **(B)** Gamma oscillation. (*N* = 7 for each state, mean ± SEM, *p < 0.05, **p < 0.01, ***p < 0.001, Kruskal–Wallis test with post-hoc pairwise comparison).

Next, we quantitatively calculated the absolute power intensity and relative power of theta and gamma oscillations. The results of each animal are shown in Supplementary Figure S1, and the statistical results are shown in Figure 3A. Our findings indicate that the absolute power of the theta frequency band significantly increase with TUS under the anesthesia and awake states, and there is no significant change in the absolute power of the theta frequency band with TUS under the running state (*N* = 7 for each state, Mean ± SEM, *p < 0.05, Kruskal–Wallis test with post-hoc pairwise comparison). These results indicate that TUS could change the absolute power of the theta frequency band under anesthesia and awake states. Since the baselines of theta absolute power in the three states are different (anesthesia: 0.7 mV^2^/Hz, awake: 10.5 mV^2^/Hz, running: 19.9 mV^2^/Hz), we calculated the relative change in absolute power to analyze the dependence of the change in theta absolute power caused by TUS on the behavioral state. The experimental results show that there are significant differences in the relative changes in absolute power after TUS between each state at 0–1 s (anesthesia: 4.6 ± 0.7, awake: 2.7 ± 0.6, running: 0.07 ± 0.6, *N* = 7 for each state, mean ± SEM, *p < 0.05, **p < 0.01, ***p < 0.001, Kruskal–Wallis test with post-hoc pairwise comparison) and 1–2 s (anesthesia: 2.1 ± 0.3, awake: 3.0 ± 0.7, running: 0.3 ± 0.1; *N* = 7 for each state, mean ± SEM, *p < 0.05, Kruskal–Wallis test with post-hoc pairwise comparison). This result indicates that the absolute power of theta caused by TUS depends on the behavioral state. The absolute power of the gamma frequency band is shown in Supplementary Figure S2 (each animal) and Figure 3B. The absolute power of the gamma frequency band increased at 0–1 s under the anesthesia and awake states and there is no significant change under the running state (*N* = 7 for each state, Mean ± SEM, *p < 0.05, Kruskal–Wallis test with post-hoc pairwise comparison). These results indicate that TUS can change the absolute power of the gamma band under anesthesia and awake states. We found that the change in absolute power in the gamma band is significantly different with TUS in the three states at 0–1 s (0–1 s, anesthesia: 3.1 ± 0.5, awake: 1.7 ± 0.4, running: 0.3 ± 0.2, *N* = 7 for each state, Mean ± SEM, *p < 0.05, Kruskal–Wallis test with post-hoc pairwise comparison). This result indicates that the absolute power of gamma frequency caused by TUS depends on the behavioral state.

**Figure 4:**
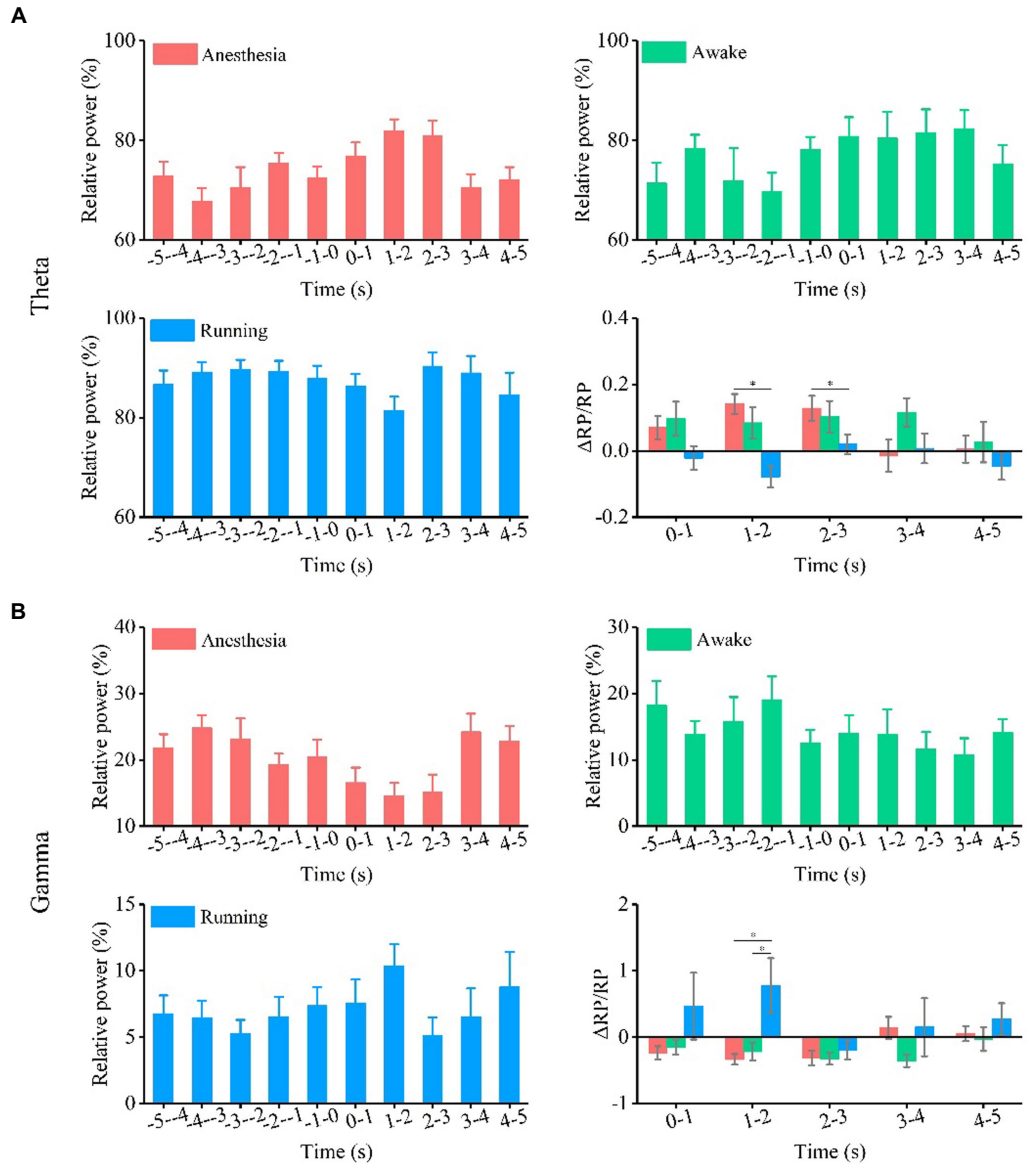
Relative power and relative change of relative power of theta and gamma oscillation under the anesthesia, awake state, and running state. **(A)** Theta oscillation. **(B)** Gamma oscillation. (*N* = 7 for each state, mean ± SEM, *p < 0.05, Kruskal–Wallis test with post-hoc pairwise comparison).

Finally, we analyzed the relative power of theta and gamma frequency bands, as shown in Supplementary Figure S2 and Figure 4. The experimental results (Figure 4A) show that the relative power of theta bands has no change trend with TUS under the three behavior states. The relative change in the relative power of theta bands is statistically significant between different behavior states at 1–2 s and 2–3 s (1–2 s, anesthesia: 0.14 ± 0.03, awake: 0.08 ± 0.05, running: −0.08 ± 0.03; 2–3 s, anesthesia: 0.13 ± 0.04, awake: 0.1 ± 0.05, running: 0.02 ± 0.03, *N* = 7 for each state, Mean ± SEM, *p < 0.05, Kruskal–Wallis test with post-hoc pairwise comparison). The results in Figure 4B indicate that the relative power of gamma bands has no significant change under the three behavior states. There is a significant difference in the relative change in the relative power of gamma between behavioral states at 1–2 s (1–2 s, anesthesia: −0.3 ± 0.08, awake: −0.2 ± 0.1, running: -0.8 ± 0.4, *N* = 7 for each state, Mean ± SEM, *p < 0.05, Kruskal–Wallis test with post-hoc pairwise comparison). The results show that TUS can significantly change the relative power of gamma under the anesthesia state, and the relative change in the relative power of the gamma frequency band depends on the behavioral state. All the above results demonstrate that ultrasound stimulation can modulate the absolute and relative power of theta and gamma bands in the CA1 and that the modulation effect depends on the behavioral state.

### 3.2. Pac between theta and gamma oscillations induced by TUS under different behavioral states

**Figure 5:**
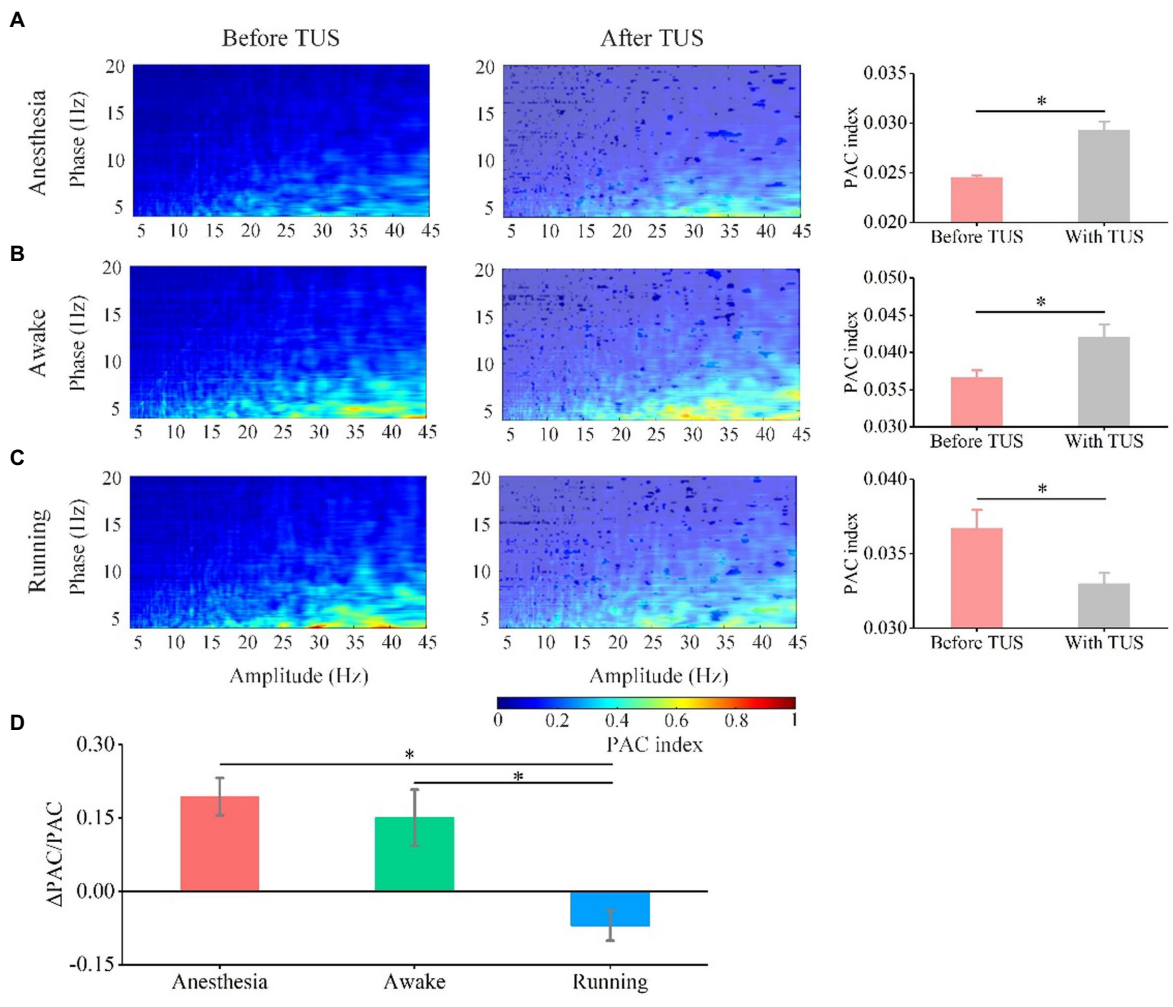
Phase-amplitude coupling between theta and gamma oscillation before and with TUS under the anesthesia, awake, and running states. **(A)** Anesthesia state. **(B)** Awake state. **(C)** Running state. **(D)** Relative change in PAC under the anesthesia, awake state, and running state. (*N* = 7 for each state, mean ± SEM, *p < 0.05, Kruskal–Wallis test with post-hoc pairwise comparison).

The PAC used to measure the modulation effect of the phase of the signal on the amplitude of this signal is considered to be the carrier mechanism of the interaction between the local and the whole of different rhythms (32, 33). Analyzing the phase-amplitude coupling relationship between theta and gamma frequency bands can reflect the distributed information integration of the brain neural network (34, 35). We analyzed the PAC between theta and gamma frequency bands to investigate the effect of behavioral state on the PAC strength induced by TUS. As shown in Figures 5A–C, we notice that the coupling strength between theta and gamma is very weak before TUS and enhanced with TUS under the anesthesia and awake states. We also notice that the theta and gamma are coupled before TUS under running state, however, interestingly, the coupling strength shows a weakening trend with TUS. We quantitatively analyzed the PAC index of each animal (Supplementary Figure S3) and the average value of all animals (Figures 5A,B). The results show that the PAC strength is significantly enhanced by TUS under the anesthesia and awake states (anesthesia: from 0.025 ± 0.0002 to 0.029 ± 0.0009, awake: from 0.036 ± 0.001 to 0.042 ± 0.0018), while there is no significant change under the running state (running: from 0.037 ± 0.001 to 0.033 ± 0.0007; *N* = 7, for each state, Mean ± SEM, *p < 0.05, **p < 0.01, Kruskal–Wallis test with post-hoc pairwise comparison). In addition to statistics PAC index, the relative change in PAC index before and with TUS was also quantitatively analyzed to evaluated the neuromodulation effect of ultrasound. The relative changes in the PAC index (ΔPAC/PAC) in Figure 5D are 0.19 ± 0.04, 0.15 ± 0.06, and − 0.07 ± 0.03 under the anesthesia, awake, and running states, respectively (*N* = 7 for each state, Mean ± SEM, *p < 0.05, Kruskal–Wallis test with post-hoc pairwise comparison). This result indicates that the PAC of theta and gamma oscillations induced by TUS are closely related to the behavioral state of animals. The above results demonstrate that TUS can modulate the PAC between theta and gamma and that the modulation effect depends on the behavioral state.

### 3.3. Relationship between relative power, PAC of theta and gamma oscillation, and ultrasound intensity under different behavioral states

**Figure 6:**
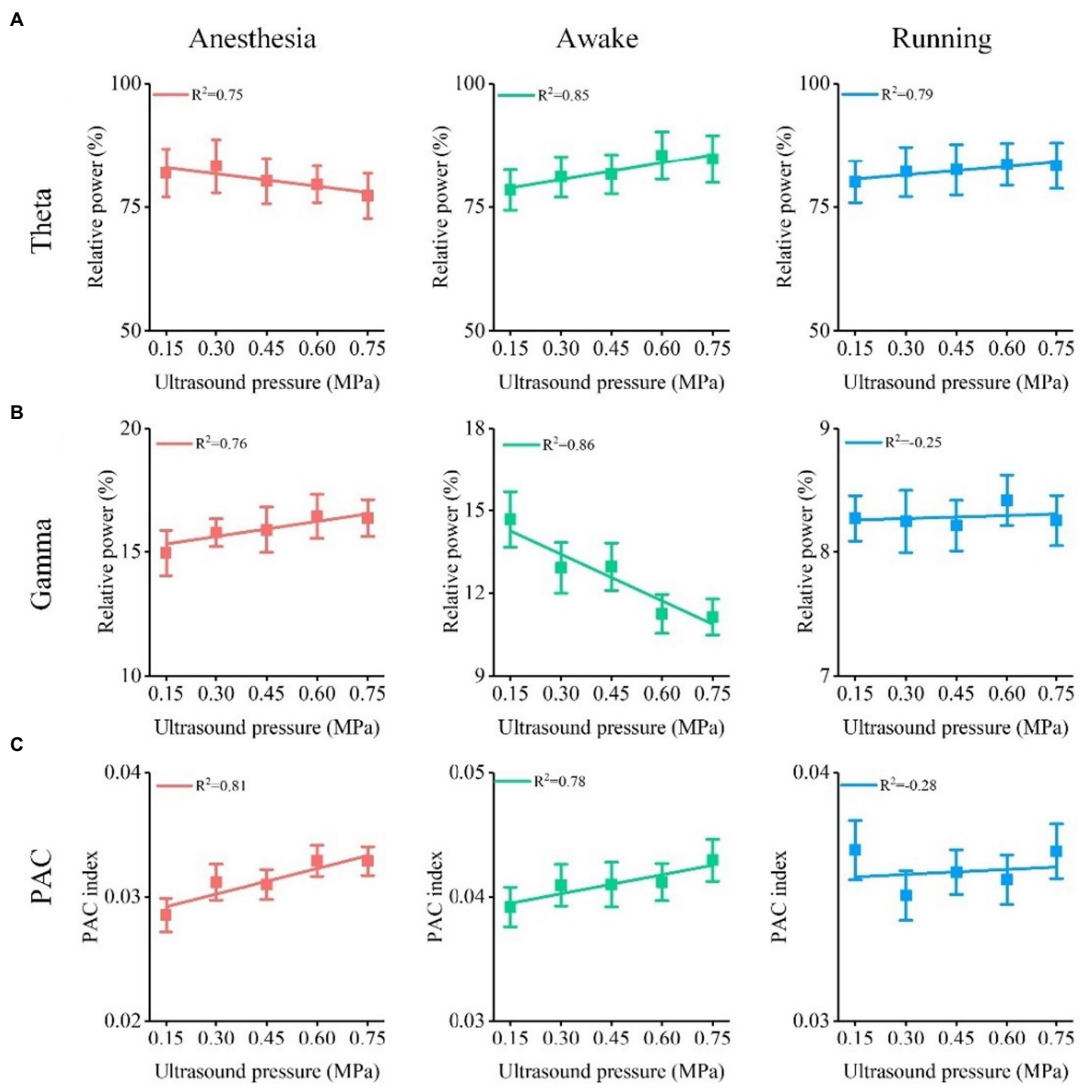
Relationship between relative power, PAC of theta and gamma oscillation, and ultrasound intensity under different behavioral states. **(A)** Relative power of theta oscillation vs. ultrasound intensity. **(B)** Relative power of gamma oscillation vs. ultrasound intensity. **(C)** ΔPAC/PAC between theta and gamma oscillation vs. ultrasound intensity.

As well-known, the ultrasound intensity plays key role in leading to different neuromodulation effects in ultrasound stimulation (5, 18, 36). We evaluated the relationship between relative power, PAC of theta and gamma oscillation, and ultrasound intensity under different behavioral states. The results in Figure 6A show that the relative power of the theta frequency band gradually decreased under the anesthesia state and gradually increased under the awake and running states with increasing ultrasound intensity. In contrast, we found that the relative power of the gamma frequency band gradually increased under the anesthesia state and gradually decreased under the awake state with increasing ultrasound intensity (Figure 6B). Similarly, we also found that the PAC strength of theta and gamma oscillations gradually increased under the anesthesia state and decreased under the awake state with increasing ultrasound intensity (Figure 6C). These results demonstrate that the theta and gamma oscillations caused by TUS are closely related to ultrasound intensity and that the correlation depends on the behavioral state.

## 4. Discussion

In this study, we used ultrasound to stimulate the CA1 region of mice and recorded the LFP before and with TUS. The analysis of the LFP demonstrated that ultrasound stimulation can modulate theta and gamma oscillations and that the modulation effects depend on behavioral states. To our knowledge, this is the first study to investigate the relationship between theta and gamma oscillations and behavioral states during TUS.

The power spectral density reflects the frequency components of the signal, as well as the distribution of the signal power at each frequency. It is commonly used in the prediction of brain neural information processing and the diagnosis of related brain diseases (37, 38). In this study, we calculated the power spectrum of the LFP and analyzed the theta and gamma frequency bands to evaluate the effect of ultrasound on theta and gamma oscillations in the CA1 under different behavioral states. We found that the absolute power of the theta and gamma frequency bands before ultrasound stimulation under running state was greater than that under awake and anesthesia states (running> awake> anesthesia). For example, the absolute powers at −1-0 s under the anesthesia, awake, and running states were 0.7 ± 0.1, 10.5 ± 3.5, and 19.9 ± 3.5 mV^2^/Hz, respectively. This indicates that the neural activity of theta oscillation gradually increased from the anesthesia to running states. The absolute power of theta with ultrasound stimulation increased significantly only under the anesthesia and awake states, while there was no significant change under the running state. The extracellular currents underlying theta waves are generated mainly by entorhinal input, CA3 (Schaffer) collaterals, and voltage-dependent Ca^2+^ currents in pyramidal cell dendrites (9). A previous study demonstrated that ultrasound stimulation altered the surface spatial morphology of the Ca^2+^ response (39). We speculate that ultrasound stimulation-modulated Ca^2+^ release is the reason for the increase in absolute power in the theta frequency band under the anesthesia and awake states. The results also showed that ultrasound stimulation weakened the intensity of theta oscillation during running. We know that when the mouse is running, the hippocampus will produce regular theta oscillations (40, 41). The reason may be that ultrasound stimulation affects neuronal activity by modulating the release of calcium ions, which breaks the original regular theta oscillations and weakens the power intensity of theta frequency bands.

We found that the absolute power intensity of theta oscillation under the running state is higher than that under the anesthesia and awake states, which means that the activity of neurons is more intense. Correspondingly, there is more Ca^2+^ release or even saturation. Even when the neurons were stimulated by ultrasound, there was no more Ca^2+^ release. Similar experimental results have also been found in other brain stimulation experiments. For example, Adam M Packer et al. applied simultaneous all-optical manipulation and recording of neural circuit activity to compare the local network response to photostimulation while mice were under the running, awake, or anesthesia state (42). For cortex cells without stimulation, they found that inferred spike responses increased from the anesthesia to awake to running states. This means that the baseline level at the awake and running states was higher than that at the anesthesia state, which is similar to results observed in CA1. In their other findings, the amplitude of the Ca^2+^ response by photostimulation increased from the anesthesia to awake to running states.

We also found that, compared with the running state, the relative change in the absolute power of gamma at 0–1 s was also significantly increased under the anesthesia and awake states. We know that the neural network composed of inhibitory interneurons is one of the main conditions for producing this high-frequency rhythmic activity. When only the fast-fired inhibitory interneurons are active at high frequency (20–80 Hz), the power of the LFP at 20–80 Hz frequency bands increases (43). Previous studies have shown that ultrasound modulation can induce action potentials of interneurons (44), and we speculate that it may be the reason for the enhancement of gamma oscillation under anesthesia and awake states. In addition, the absolute power of gamma does not significantly change under the running state. It may be that neuron excitement is high under the running state, and ultrasound stimulation cannot further enhance neuron excitement.

As we know, the power spectrum interpretations can provide the energy intensity of neural oscillations (45, 46). We have observed that ultrasound stimulation can significantly modulate power spectrum of LFP under anesthesia and awake states. This result means that, compared with the running state, ultrasound stimulation is easier to alter the excitability of neurons under anesthesia and awake states, which provides guidance to choose the state of the sample for ultrasound stimulation.

In our study, we noticed that ultrasound stimulation induces an increase in the strength of the phase amplitude coupling between theta and gamma under anesthesia and awake states. When mice are under anesthesia, their brain neurons have less functional activity, and the phase amplitude coupling between theta and gamma is weak. However, when neurons are stimulated by ultrasound, the neuron group will conduct a self-organizing discharge response according to ultrasound stimulation, including an increase in theta rhythm and gamma rhythm, which will result in a more obvious PAC reaction. When awake mice are stimulated by ultrasound, their neuronal activity will still show stronger responses, such as the enhancement of theta oscillation and the coupling strength of theta and gamma. We also found that the PAC strength between theta and gamma is weakened under the running state. One possible reason is that there is strong phase amplitude coupling between theta and gamma before TUS. Neural activity was disrupted by external stimuli, such as a decrease in theta oscillation, thereby reducing the coupling strength between theta and gamma.

In this study, we found that the relationship between theta, gamma oscillations caused and ultrasound intensity depends on the behavioral state. As we know, the theta waves are generated mainly by the entorhinal input, CA3 collaterals, and voltage-dependent Ca^2+^ currents in pyramidal neuron (excitatory neuron) dendrites (8). A network of inhibitory interneurons is a key factor in the generation of gamma rhythm oscillations (43, 47). The theory of ultrasound stimulation based on the cavitation effect predicted that ultrasound stimulation is selective for neurons. Recent experimental research, which shows that excitatory and inhibitory neurons are intrinsically different in response to ultrasound pulse repetition frequency, supports the above theoretical speculation. In addition, studies have also shown that the success rate of motion response and hemodynamic response caused by ultrasound stimulation are closely related to ultrasound intensity. Therefore, we speculate that the selectivity of neurons under ultrasound stimulation causes the opposite trend of relative power of theta and gamma with the increase of ultrasound intensity under anesthesia and awake states. Indeed, oscillations in the hippocampal LFP of mouse/rat at theta and gamma frequencies are prominent during running behavior (48, 49). However, there are no significant theta and gamma oscillations under anesthesia and awake (fixed head without free movement) states. The excitement of neural activity in the hippocampal LFP under running state is higher than that under anesthesia and awake state. Previous studies demonstrated that the excitement of neural activity affects the hemodynamic response caused by visual stimulation (50) and the firing rate of action potential by photostimulation (42). Therefore, we speculate that the excitement of neural activity may cause the neuromodulation of TUS under running states were different from those under anesthesia and awake states. We will conduct in-depth research to verify the above potential mechanisms in our next work.

Guo et al. (51) and Sato et al. (52) found that the cortical neural activities was induced by TUS with non-specific auditory responses. However, these results are countered some recent work. Mohammadjavadi et al. (53) demonstrated that direct activation of central motor neural circuits of deaf knockout mice is *via* ultrasound stimulation rather than *via* a startle reflex by auditory responses. In our previous study, we found that TUS suppressed parkinsonian-related activity by stimulating brain tissue, not by auditory effects (26). Recently, a novel work by Yu et al. demonstrated that the directly local neural effects induced by ultrasound stimulation without potential auditory confounds with chemically deafened rats and genetically deafened mice (54). Therefore, we believe that our study putatively presents directly ultrasound-mediated neural activity responses in hippocampus, however, future research is needed to further clarify auditory responses.

In this study, since we fixed the mouse head to perform experiments for awake mice, and controlled the treadmill to keep the mice under running state, it is an awake or a running model with stress. In previous studies, researchers designed headmounted ultrasound transducers for ultrasound neuromodulation in awake and freely moving mice/rat (55–58). They achieved superior neuromodulation effects for healthy or Parkinson’s mice. However, due to technical limitations, we did not have a wearable miniaturized ultrasound transducer in our laboratory. Therefore, we were unable to carry out ultrasound stimulation experiments for freely moving animals. We will try to develop the miniaturized ultrasound transducer for modulating freely moving animals.

Because behavioral states represent completely different levels of neural excitement, studying the LFP induced by TUS under different behavioral states can reflect the behavioral state dependence of TUS and provide a basis for ultrasound stimulation. Our research shows that TUS can modulate theta and gamma oscillations in CA1 and that the modulation effect strictly depends on the behavioral state; however, the exact mechanism is still unclear. We will carry out mechanistic research in future work.

## 5. Conclusion

In summary, our results demonstrate that (1) TUS significantly enhanced the absolute power of theta and gamma oscillations under the anesthesia and awake states. (2) The PAC between theta and gamma oscillations was significantly enhanced under the anesthesia and awake states but was significantly weakened under the running state by TUS. (3) Under the anesthesia state, the relative power of theta decreases and that of gamma increases as the ultrasound intensity increases, and the result under the awake state is opposite that under the anesthesia state. (4) The PAC index between theta and gamma increases as ultrasound intensity increases under the anesthesia and awake states and decreases under the running state. This study has great potential benefits for the application of ultrasound stimulation in cognitive neuroscience and the treatment of neuropsychiatric diseases.

## Data availability statement

The original contributions presented in the study are included in the article/Supplementary material, further inquiries can be directed to the corresponding author.

## Ethics statement

The animal study was reviewed and approved by the Animal Ethics and Administrative Council of Capital Medical University.

## Author contributions

ZL designed and coordinated the study. RC, DL, XW, and WY carried out experiment and data process, and drafted the manuscript. All authors contributed to the article and approved the submitted version.

## Conflict of interest

The authors declare that the research was conducted in the absence of any commercial or financial relationships that could be construed as a potential conflict of interest.

## Publisher’s note

All claims expressed in this article are solely those of the authors and do not necessarily represent those of their affiliated organizations, or those of the publisher, the editors and the reviewers. Any product that may be evaluated in this article, or claim that may be made by its manufacturer, is not guaranteed or endorsed by the publisher.
