# Supplementary material for: Effect of low-intensity transcranial ultrasound stimulation on theta and gamma oscillations in the mouse hippocampal CA1
Source: Front Psychiatry. 2023 Apr 20;14:1151351. doi: 10.3389/fpsyt.2023.1151351 (PMC10157252; doi:10.3389/fpsyt.2023.1151351)
Supplement: Supplementary file 1 [file Data_Sheet_1.docx]

**Effect of low-intensity transcranial ultrasound stimulation on theta and gamma oscillations in the mouse hippocampal CA1**

Zhen Li^1,#,*^, Rong Chen^2,#^, Dachuan Liu^1^, Xizhe Wang^1^, Wei Yuan^1^

^1^Department of Ophthalmology, Xuanwu Hospital, Capital Medical University, Beijing 100053, China

^2^Hebei Key Laboratory of Vascular Homeostasis and Hebei Collaborative Innovation Center for Cardio-cerebrovascular Disease, the Second Hospital of Hebei Medical University, Shijiazhuang, 050000, China

^#^These authors have contributed equally to this work

*Address corresponding to Zhen Li, E-mail: [zhenli_xwyy@163.com](mailto:zhenli_xwyy@163.com)


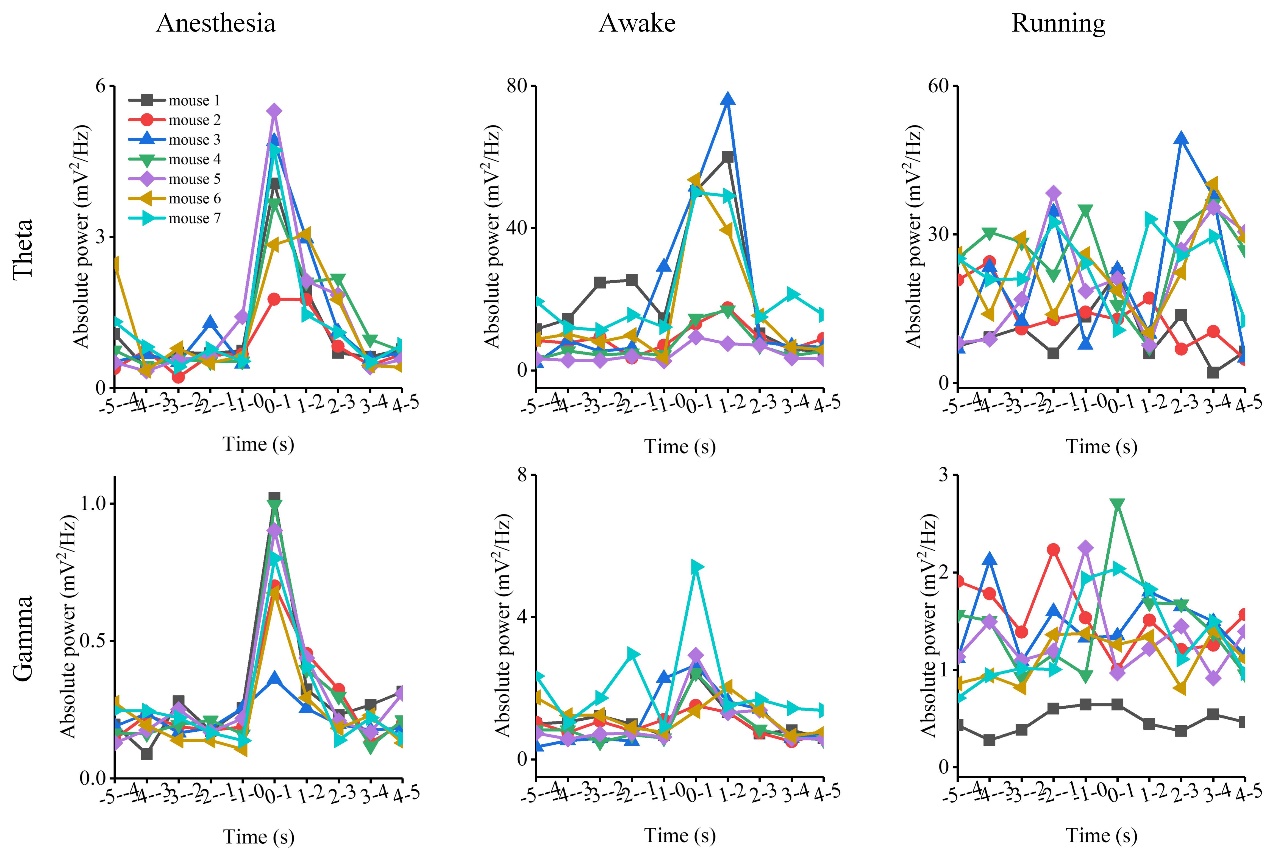


**Figure S1.** Absolute power of theta and gamma oscillation for each animal under the anesthesia, awake state, and running state.


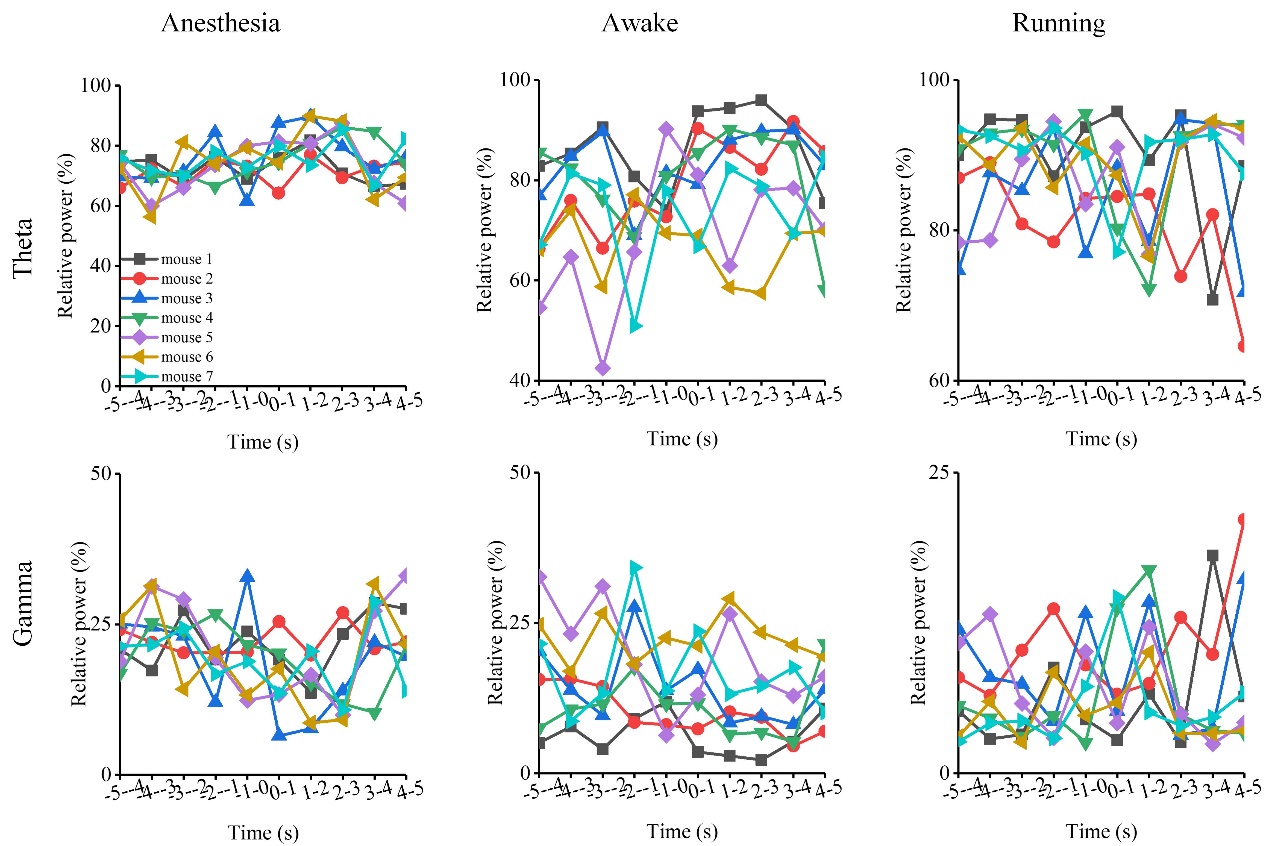


**Figure S2.** Relative power of theta and gamma oscillation for each animal under the anesthesia, awake state, and running state.


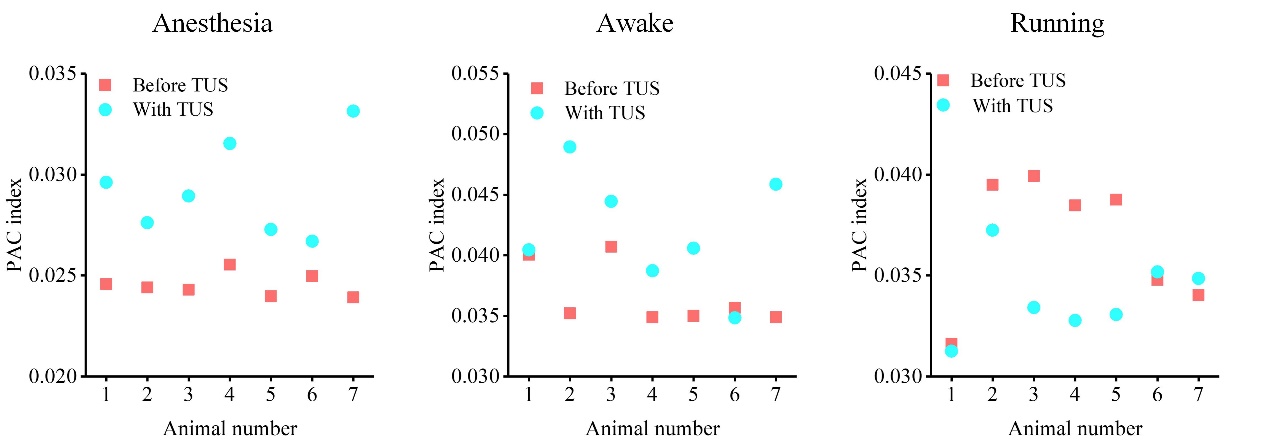


**Figure S3.** Phase-amplitude coupling between theta and gamma oscillation before and after TUS for each animal under the anesthesia, awake, and running states.
